# Defining the Role of Anaortic Coronary Artery Bypass Grafting

**DOI:** 10.3390/jcm12144697

**Published:** 2023-07-14

**Authors:** Fabio Ramponi, Michael Seco, Michael P. Vallely

**Affiliations:** 1 Department of Cardiovascular Surgery, Mount Sinai Morningside, New York, NY 10025, USA; fabio.ramponi@mountsinai.org; 2 Department of Cardiothoracic Surgery, Royal North Shore Hospital, Sydney 2065, Australia; 3 Department of Cardiothoracic Surgery, Monash Health, The Victorian Heart Hospital, Melbourne 3168, Australia; 4 Department of Surgery, Monash University, Melbourne 3168, Australia

**Keywords:** coronary surgery, off-pump, anaortic, stroke

## Abstract

As the population ages and co-morbidities become more prevalent, the complexity of patients presenting for coronary artery bypass surgery is increasing. Cardiopulmonary bypass and aortic cross-clamping in these patients carry increased risk and, indeed, in some patients, with ascending aortic disease, the risks are prohibitive. Total-arterial anaortic coronary artery surgery is a technique that provides complete surgical coronary artery revascularization without cardiopulmonary bypass and without manipulating the ascending aorta. The technique essentially eliminates the risk of cerebral embolization of aortic atheroma and aortic injury. Anaortic techniques are an essential skillset for coronary artery surgery centers treating higher-risk patients.

Anaortic coronary artery bypass grafting is a technique of off-pump surgical coronary artery revascularization that completely avoids aortic manipulation by using composite grafts with in-flow from one or both IMAs or the gastro-epiploic artery, often using all-arterial grafts. Typically, the IMAs are used for in-flow, and our group has published on the use of a single IMA and double IMAs as the in-flow for the radial artery as a composite T or I graft, respectively [1,2].

There are patients for whom the use of cardiopulmonary bypass and aortic cross-clamping confers such a risk that its use is absolutely contraindicated. These patients include but are not limited to those with a porcelain ascending aorta and/or grade V mobile atheroma in the ascending aorta or aortic arch (Figure 1, Panel 1a and 1b, and Panel 2a and 2b). These patients have a prohibitive risk of disseminated emboli from the aortic cross-clamp, the jet from the aortic cannula inflow and, in the case of a porcelain aorta, aortic rupture and or dissection from a cross-clamp injury [3].

To define the role of anaortic or “aortic not touch” coronary surgery, it is now widely accepted that the technique confers the greatest clinical benefit to patients at high risk for aortic atheroembolism because of aortic manipulation during CABG from aortic cannulation, cross-clamping, and the placement of aorto-coronary grafts on the ascending aorta [4,5]. Indeed, anaortic surgery carries a Class 1b indication in the EACTS/ECC 2018 Coronary Revascularization Guidelines for patients with a diseased ascending aorta [6] (Figure 2, Panel 1). Similarly, a joint statement from the American Heart Association and The American Stroke Association in 2021 established that Anaortic OPCAB was the standard of care in the elimination of embolic stroke after CABG [7] and is a Class 2a indication in the most recent AHA coronary guidelines [8].

However, there is increasing evidence to show that embolic strokes, as defined by new lesions on diffusion-weighted MRI [9], occur in up to 40% of patients undergoing on-pump coronary artery bypass surgery with aortic cross-clamping [9]. It has also been established that occult strokes are not benign and lead to significantly higher short- and long-term morbidity and mortality [9,10]. Therefore, there may be a compelling argument for a more widespread use of anaortic techniques in the performance of routine CABG.

The CORONARY trial is widely regarded as the most robust randomized controlled trial comparing on-pump to off-pump CABG in higher-risk cases, performed by experienced surgeons [11]. The CORONARY trial, however, failed to demonstrate a neurological benefit of OPCAB over on-pump CABG. The likely reason why the CORONARY trial failed to show a benefit was that the OPCAB technique was not specified or reported in the results. Some patients had proximal anastomoses performed using a side-biting clamp, others were treated with a proximal anastomotic device, and some patients had anaortic surgery. Therefore, it was not possible to demonstrate the benefit of anaortic surgery in the setting of a surgical randomized control trial.

In an effort to establish the evidence for reducing aortic manipulation during CABG, a network meta-analysis of more than 46,000 patients published by our group in 2017 demonstrated a clear neurological benefit of completely avoiding aortic manipulation during CABG, with a 78% stroke reduction compared to on-pump CABG [4] (Figure 2, Panel 2). More recently, a randomized controlled trial of OPCAB with and without aortic manipulation showed a three-fold reduction in post-operative delirium and cognitive dysfunction when anaortic surgery was compared to a side-biting clamp on the ascending aorta [12].

The technical details of the performance of anaortic OPCAB have been described in several publications by our group [1,13]. There are three potential arterial inflows for the composite grafts. These are the left and right internal mammary arteries and the gastroepiploic artery. Our group has limited experience with the gastroepiploic artery which is more commonly used in Japan and other Asian countries, with excellent published outcomes [14].

We prefer using two IMA inflows, with a LIMA to the LAD and a RIMA/radial artery composite “I” graft, via the transverse sinus, using multiple sequential distal anastomoses to targets on the lateral and inferior walls. This offers separate inflows, maintaining the integrity of the LIMA to the LAD, reducing the risk of competitive flow away from the LAD. There is also a theoretical benefit of increased blood flow for exercise. There is some evidence to show that long-term survival is greater with two IMA in-flows compared to a single LIMA in-flow [15]; however, other authors have demonstrated excellent long-term outcomes for single-IMA/radial composite Y/T grafts [16].

Concerns regarding deep sternal wound infection and the use of BIMAs have been used by some as a reason not to pursue this technique. However, data from the ART trial [17] and our own series [18] support the routine use of BIMA in CABG if the IMAs are harvested using a skeletonized technique. Skeletonized arteries are also longer, often easier to use, and offer flexibility for the construction of composite grafts and the performance of sequential grafts. There is also evidence for less bleeding and need for transfusion if the IMAs are taken in a skeletonized fashion [19].

The use of composite grafts allows for significant flexibility in the deployment of grafts, and the common configurations are demonstrated in Figure 3 (Panel 1). The mainstay of our technique remains the two IMA inflows with a RIMA/RA tandem/I graft. However, several researchers [16] have published on the use of a single LIMA in-flow with RA composite T or Y grafts. Both techniques are easily reproducible when the surgeon and the wider team have sufficient training and become proficient. The use of composite grafts has been shown to be equivalent, if not superior, to the use of aorto-coronary grafting [20,21]. Another advantage of composite grafts is the ability to perform more grafts, often increasing the number of arterial grafts and decreasing or eliminating the use of vein grafts [22]. The conduit harvesting time is reduced, and there is also a need for fewer proximal anastomoses.

Cardiac positioning is facilitated using pericardial release incisions down to the SVC at the SVC/RA junction and at the diaphragm/IVC junction. Care must be taken to avoid a phrenic nerve injury when performing these release incisions (Figure 3, Panel 2). Placing the left-sided pericardium behind the sternal retractor also delivers the heart anteriorly and creates more space to observe the lateral wall when grafting. The use of silastic intracoronary shunts is also imperative. This not only provides a relatively bloodless field and distal coronary perfusion but also makes catching the back wall of the coronary artery virtually impossible, therefore minimizing technical mishaps. The use of TTFM flow probe assessment is also an essential quality control measure in all coronary surgery scenarios [23].

There are some patients for whom off-pump surgery is not possible or its performance compromises the perfusion of other organs. Cardiomegaly can be problematic when positioning the heart in order to graft the lateral wall. Some hearts dilate in a longitudinal fashion, and the procedure can be assisted with a more aggressive “verticalization” of the heart to facilitate lateral wall grafting. Some hearts dilate in the transverse plane, and it can be almost impossible to deliver the lateral wall safely to facilitate grafting. In our experience, this scenario is most common in obese patients with diabetes and poorly controlled hypertension.

If conversion to on-pump surgery is required, then it is important that this is managed in a controlled fashion. We have a relatively simple approach to this situation. If the positioning of the heart is not possible, despite all anesthetic and OR table maneuvers having been tried, then we stratify the patients into those in whom the risk of aortic cross clamping is unacceptable and those in whom it is not. Beating heart on-pump is a good strategy in cases where the conversion to on-pump surgery is for ischemia; however, when it is for cardiomegaly, the heart may not decompress enough to be easily positioned. In this situation, we may use the traditional aortic cross-clamping, cardioplegic arrest, and left ventricular venting technique.

As the population ages and the complexity of intercurrent co-morbidities increases, cardiovascular interventionalists are facing increasing challenges to deliver appropriate therapy to this group of patients. Patients with severe structural heart disease, such as aortic stenosis or mitral regurgitation, often have intercurrent coronary artery disease. The severity of atherosclerosis and vascular calcification can mean that coronary stents are inappropriate or have been used in the past and have now failed and that coronary disease requires surgical correction. This can be managed with anaortic CABG techniques, as described above and elsewhere [3]. However, a significant challenge is when the patient also has structural heart disease of a severity that precludes a staged approach.

Our group developed a program of concurrent anaortic OPCAB and TAVR during the same anesthetic, performed in a hybrid operating room. The anaortic OPCAB is performed first via median sternotomy or a left-anterior thoracotomy. The heparin is reversed, and the patient is then closed, re-prepped, and draped, and the transfemoral TAVR is performed in the routine fashion. This allows for the concurrent safe treatment of complex coronary artery disease and severe aortic stenosis, peri-operatively avoiding cardiac ischemia or aortic stenosis related low cardiac output. We recently published a series of eight patients, including technical considerations [24].

Similarly, patients with severe mitral regurgitation in the setting of a porcelain circulation may undergo anaortic OPCAB and then have the mitral valve treated with percutaneous edge-to-edge repair. However, the challenges of positioning the heart for OPCAB in the setting of severe mitral regurgitation are not insignificant. A more reasonable approach may be to treat the mitral valve with a Mitra-Clip at least three months prior to anaortic OPCAB. This would allow for the endothelialization of the Mitra-Clip and perhaps a reduction in left ventricular size, thus preventing clip dislodgement and assisting in cardiac positioning during OPCAB, respectively.

Patients with complex cerebrovascular and peripheral vascular disease often present for surgical coronary revascularization. We reported on a series of 39 combined carotid endarterectomy and anaortic CABG with 5.2% mortality and a 2.6% stroke rate [25]. The carotid endarterectomy was performed first by a specialist vascular surgeon and then anaortic OPCAB was performed by an anaortic specialist. More recently, our group presented the results of a network meta-analysis which established that a combined carotid endarterectomy and OPCAB approach resulted in superior neurological and cardiac outcomes over a staged or reverse-staged approach [26].

Coronary artery bypass surgery remains the most frequently performed operation in adult cardiac surgery. It is a procedure that is often viewed as a commodity item and not as a sub-specialist procedure. The sheer volume of patients requiring the procedure and the fact that it is often performed in smaller centers in a sub-acute setting perpetuates this. However, there is a need for this specialty to recognize the role that advanced coronary artery surgery plays in the delivery of care for patients, especially in the context of the ageing population.

Today, coronary artery surgery is being recognized as a heterogenous technique, where the use of more advanced techniques such as all-arterial and anaortic approaches may yield superior shorter- and longer-term outcomes. Traditionally, surgeons have been reluctant to refer patients on to sub-specialist surgeons for fear of losing their own practice. However, it behooves us as a craft group to recognize that not all patients can be treated safely by all surgeons and/or centers, and there needs to be mechanisms with which to recognize complex patients and have them referred on appropriately.

A pragmatic approach to more advanced techniques in cardiac surgery needs to be accepted. Not all surgeons are comfortable with all techniques. As highlighted above, the advantages of anaortic surgery are most relevant in patients with severe aortic disease. A simple approach is to pre-operatively screen all-comers with a non-contrast CT and to triage the patients to the appropriate surgeon within the unit and to refer outside the unit if the expertise does not exist within the unit. In our practice, we perform a non-contrast CT chest for all-comers, with the exception of very young patients (i.e., <50 yo) and unstable patients for whom transfer to a CT scanner would be unsafe. We perform epi-aortic US for all patients regardless of the revascularization technique.

Across the spectrum of cardiac surgery, high-volume surgeons and high-volume centers have been shown to achieve better outcomes than their lower-volume counterparts [27,28]. Surgery is a technical exercise, and there are subtleties to each patient that require the surgeon and the wider team to make decisions of a technical and management nature that will affect the outcome. OPCAB is widely accepted to be a technically more difficult procedure than on-pump CABG, and to this end, volume and expertise will more than likely confer significant outcome benefits for patients [29].

Individual surgeon experience in OPCAB is an important determinant in patient selection for OPCAB. The unique technical challenges of OPCAB grafting (especially anaortic techniques) and its relative unfamiliarity have raised concerns that the adoption of OPCAB may lead to poorer outcomes during each surgeon’s “learning curve.” With careful patient selection, OPCAB surgery can be gradually assimilated into clinical practice while preserving and ultimately improving clinical outcomes. Very early in a surgeon’s experience, it is reasonable to exclude patients with depressed left ventricular function and left main disease and those requiring multiple lateral wall grafts. With experience, more complex and technically challenging cases can be performed safely off-pump; a step-wise approach is recommended to take an individual surgeon and a surgical program from the “basic” LIMA/SVG on-pump CABG to more advanced techniques including, inter alia, total-arterial anaortic OPCAB [30]. To facilitate the safe implementation of advanced coronary surgery techniques, especially during the initial “ learning curve”, a dedicated period of fellowship training should be devoted to learning those complex procedures under the mentorship of expert surgeons and in a safe environment of specialist teams [31].

Whilst anaortic OPCAB may be more technically demanding than on-pump CABG, it is not so by a substantial or unreasonable margin. It is a reproducible technique, and it is a teachable technique (Figure 4, Panel 1). We adopted the routine use of the all-arterial anaortic OPCAB approach for all-comers. The primary reason for this is that we believe, as supported by evidence, that the technique offers superior short-term neurological benefits afforded by eliminating aortic manipulation [4] and the superior long-term benefits of all-arterial grafting [32]. A secondary and possibly equally important reason is that the technique becomes routine for the surgeon and the team. This results in technical proficiency and the ability to easily manage complex patients (Figure 4, Panel 2).

**Figure 3:**
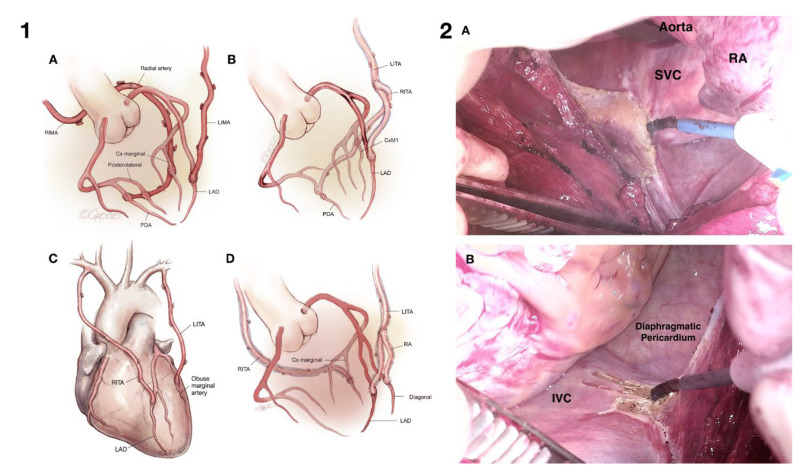
(**Panel 1**): Common configurations of composite arterial grafts. (**A**) LITA to LAD and RITA-LRA extension though the transverse sinus to the lateral and inferior system [1]; (**B**) LITA to LAD and RITA as a Y graft from LITA to the lateral and inferior systems [33]; (**C**) in situ RTA to LAD and LITA to obtuse marginal [34]; (**D**) LITA to LAD, LRA as a Y graft from LITA to diagonal branch and RITA to obtuse marginal [33]. Figures reprinted according to CC BY-NC-ND 4.0 license. (**Panel 2**): (**A**) Right superior pericardial slit. During a brief period of apnea, a vertical pericardial slit is made with diathermy down to and including the pericardial fold at the right atrial/SVC junction. The assistant retracts the thymus with their right hand using the Yankeur sucker head and retracts the aorta using reversed De Bakey forceps in their left hand. Extreme care must be taken not to injure the right phrenic nerve. (**B**) Right inferior pericardial slit. A vertical pericardial incision is made with diathermy down to the IVC. Care is taken to remain extra-pleural and to avoid injury to the right phrenic nerve [35]. Figures reprinted according to CC BY-NC-ND 4.0 license. (LITA: left internal thoracic artery; RITA: right internal thoracic artery; LRA: left radial artery).

Finally, a subgroup of patients that might benefit less from an anaortic all arterial OPCAB include patients with cardiomyopathies requiring additional interventions [36].

Although a detailed description of total endoscopic coronary artery bypass (TECAB) techniques is beyond the scope of this paper, TECAB is safely adopted in specialized centers to provide multivessel revascularization either as a single procedure or as part of a hybrid strategy [37,38]. The appeal of off-pump anaortic robotic TECAB is to obtain the long-term benefit of IMA grafting while limiting invasiveness by minimizing the surgical incisions with the aid of robotic technology. The avoidance of sternotomy and CPB has led to a reduction in post-operative length of stay (down to 2.7 days in some series) and an earlier return to normal activity and work. The application of OPCAB to patients with multivessel disease is also performed in combination with the percutaneous coronary intervention (PCI) of other territories by combining minimally invasive LIMA-LAD grafting with the PCI of non-LAD vessels. Hybrid procedures are being rigorously evaluated to determine what subpopulation of patients with multivessel disease may benefit from this approach. Patients with multivessel disease currently being treated with percutaneous techniques alone represent a group for whom hybrid procedures may be increasingly used. A recent propensity-matched analysis of the New York cardiac surgery and percutaneous interventions registries showed no difference in 6-year mortality between hydride coronary revascularization (HCR) and conventional CABG, although the latter group were more likely to be free from repeat revascularization [39].

In conclusion, the role of anaortic surgery can be defined as a necessary tool in the kit of all surgeons who wish to undertake advanced coronary artery surgery. All major units need an advanced coronary team and need to maintain good relationships with surrounding, smaller units to facilitate appropriate care for their more complex patients.

Coronary surgery and the management of ischemic heart disease need to emerge as a genuine sub-specialty with the appropriate training and recognition of expertise in the field by surgeons and referring physicians alike. The ageing population, increasing complexity of patients, and the increased scrutiny of, and expectations for, cardiac surgery demands it.

## Figures and Tables

**Figure 1:**
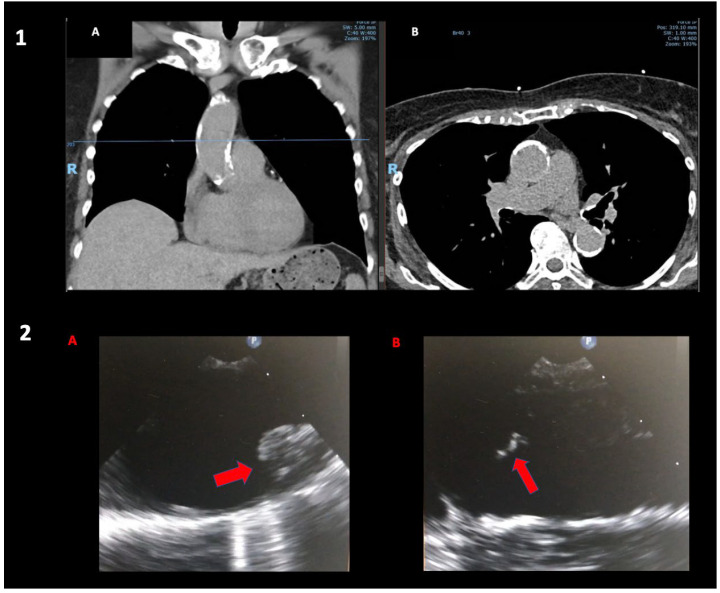
(**Panel 1**): Coronal (**A**) and axial (**B**) views of a chest CT showing extensive ascending aortic calcifications in a 75-year-old lady admitted with unstable angina; coronary angiogram showed severe distal left main disease. (**Panel 2**): Intraoperative TOE showing grade IV (>5 mm) (**A**) and grade 5 (mobile) (**B**) aortic arch atheroma of a 72-year-old man undergoing combined right carotid endarterectomy and anaortic OPCAB.

**Figure 2:**
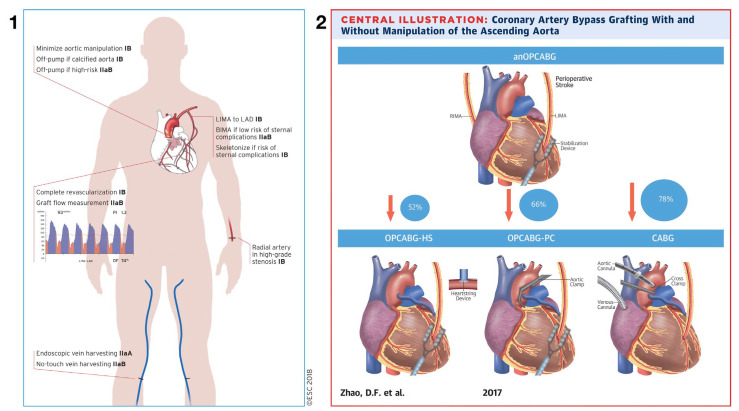
(**Panel 1**): The 2018 ESC/EACTS Coronary Revascularization Guideline recommendations for specific surgical techniques [6]. Reprinted with permission. (**Panel 2**): Four surgical methods of coronary artery bypass grafting with increasing degrees of aortic manipulation. Percentages pictured represent the relative decrease in risk of perioperative stroke using anOPCABG compared to the other techniques [4]. Reprinted with permission. (CABG: coronary artery bypass grafting with cardiopulmonary bypass; anOPCABG: anaortic off-pump coronary artery bypass grafting; LIMA: left internal mammary artery; OPCABG-HS: off-pump coronary artery bypass grafting with the Heartstring system; OPCABG-PC: off-pump coronary artery bypass grafting with partial clamp; RIMA: right internal mammary artery).

**Figure 4:**
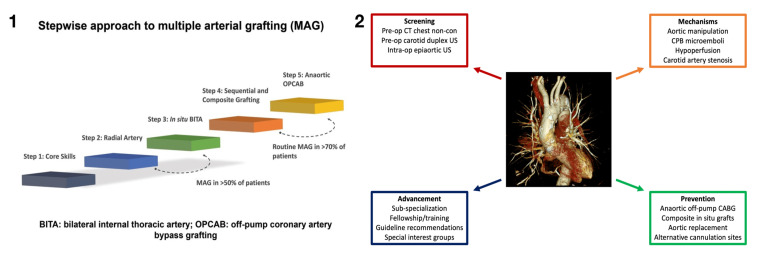
(**Panel 1**): Stepwise progression to multiple arterial grafting from the basics of acquiring the core skills to total arterial anOPCAB [30]. Reprinted with permission. (**Panel 2**): Integrated approach to reducing neurologic injury during surgical coronary revascularization [3]. Reprinted with permission. (MAG: multiple arterial grafting; anOPCAB: anaortic off-pump coronary artery bypass grafting; BITA: bilateral internal thoracic artery; CABG, coronary artery bypass grafting; CPB, cardiopulmonary bypass; CT, computed tomography.)
